# In utero human intestine harbors unique metabolome, including bacterial metabolites

**DOI:** 10.1172/jci.insight.138751

**Published:** 2020-11-05

**Authors:** Yujia Li, Jessica M. Toothaker, Shira Ben-Simon, Lital Ozeri, Ron Schweitzer, Blake T. McCourt, Collin C. McCourt, Lael Werner, Scott B. Snapper, Dror S. Shouval, Soliman Khatib, Omry Koren, Sameer Agnihorti, George Tseng, Liza Konnikova

**Affiliations:** 1 Department of Biostatistics and; 2 Department of Immunology, University of Pittsburgh, Pittsburgh, Pennsylvania, USA.; 3 Azrieli Faculty of Medicine, Bar Ilan University, Safed, Israel.; 4 Analytical Chemistry Laboratory, Tel-Hai College, Upper Galilee, Israel.; 5 Department of Pediatrics, Yale University, New Haven, Connecticut, USA.; 6 Department of Pediatrics, University of Pittsburgh, Pittsburgh, Pennsylvania, USA.; 7 Pediatric Gastroenterology Unit, Edmond and Lily Safra Children’s Hospital, Sheba Medical Center, Ramat Gan, Israel.; 8 Sackler Faculty of Medicine, Tel Aviv University, Tel Aviv, Israel.; 9 Division of Gastroenterology, Hepatology and Nutrition, Boston Children’s Hospital, Boston, Massachusetts, USA.; 10 Department of Natural Compounds and Analytical Chemistry, Migal Galilee Research Institute, Kiryat Shmona, Israel.; 11 Department of Neurosurgery and; 12 Department of Developmental Biology, University of Pittsburgh, Pittsburgh, Pennsylvania, USA.; 13 Division of Reproductive Sciences and; 14 Program in Human and Translational Immunology, Yale University, New Haven, Connecticut, USA.

**Keywords:** Gastroenterology, Metabolism, Intermediary metabolism

## Abstract

Symbiotic microbial colonization through the establishment of the intestinal microbiome is critical to many intestinal functions, including nutrient metabolism, intestinal barrier integrity, and immune regulation. Recent studies suggest that education of intestinal immunity may be ongoing in utero. However, the drivers of this process are unknown. The microbiome and its byproducts are one potential source. Whether a fetal intestinal microbiome exists is controversial, and whether microbially derived metabolites are present in utero is unknown. Here, we aimed to determine whether bacterial DNA and microbially derived metabolites can be detected in second trimester human intestinal samples. Although we were unable to amplify bacterial DNA from fetal intestines, we report a fetal metabolomic intestinal profile with an abundance of bacterially derived and host-derived metabolites commonly produced in response to microbiota. Though we did not directly assess their source and function, we hypothesize that these microbial-associated metabolites either come from the maternal microbiome and are vertically transmitted to the fetus to prime the fetal immune system and prepare the gastrointestinal tract for postnatal microbial encounters or are produced locally by bacteria that were below our detection threshold.

## Abstract 2

A unique human fetal metabolomic intestinal profile is reported with an abundance of bacterially derived metabolites.

## Graphical abstract

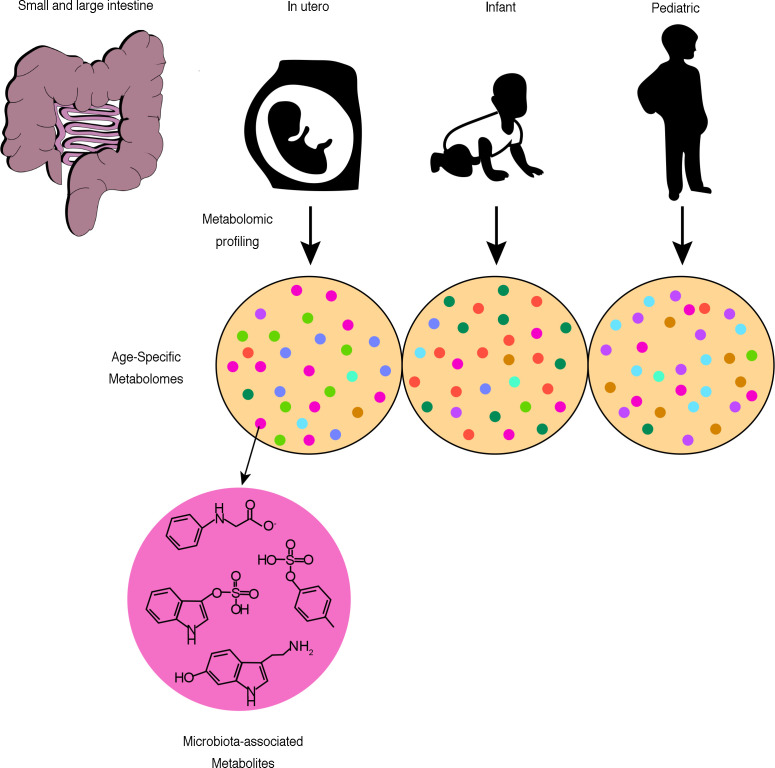

## Introduction

Early-onset sepsis (<7 days of life in healthy infants and <72 hours in NICU-resident neonates) is estimated to occur in as many as 1:1000 live births (1). Pathogens contributing to neonatal sepsis are microbes commonly vertically transmitted from the mother before or during delivery (1). Although the presence of sepsis in newborns is alarming, most neonates do not acquire bacteremia, though they may encounter similar pathogens during or after labor. It is now appreciated that education of the immune system may be ongoing in utero through the detection of memory T cells within the primate fetal intestines (2–4), cord blood (5), and placenta (6). These findings suggest that perhaps it is insufficient in utero priming to microbial products rather than improper fetal encounter with microbes that leads to bacteremia and subsequent early-onset sepsis.

The existence of an in utero microbiome remains controversial. Multiple studies have indicated that the placenta is devoid of a microbiome (7–11), whereas others have reported limited bacterial colonization in fetal meconium (12) and placental and endometrial samples (13–18). A study by Aagaard et al. (13) analyzing a large number of patients suggested that the placenta has a unique microbiome that is similar to the oral microbiome of nonpregnant women. Conversely, work by Lauder et al., also assessing a similar large cohort, compared placental samples with kit samples and found no difference between them (8).

Murine work suggests that maternally derived bacterial byproducts can be found in murine fetal intestines, drive immune and epithelial development and likely decrease postnatal pathogen translocation (19). Furthermore, human data show that malaria-specific fetal T cell responses can be generated during pregnancy and confer protection later in life (20). Collectively, this work led us to hypothesize that microbial products or metabolites may similarly be detected in the human fetal intestine and drive in utero immune development and education. Additionally, we hypothesized that the intestinal metabolomic profile of fetal, infant, and pediatric intestines is age-group specific. In this study, we were unable to detect an intestinal microbiome with 16S sequencing in fetal samples. However, we found a metabolomic fetal intestinal profile with an abundance of bacterially derived metabolites and aryl hydrocarbon receptor (AHR) ligands implicated in mucosal immune regulation (21). Consistent with murine data, these metabolites are potentially critical to the in utero immune priming and preparation of intestine for homeostasis with the postnatal microbiome.

## Results

**Figure 1:**
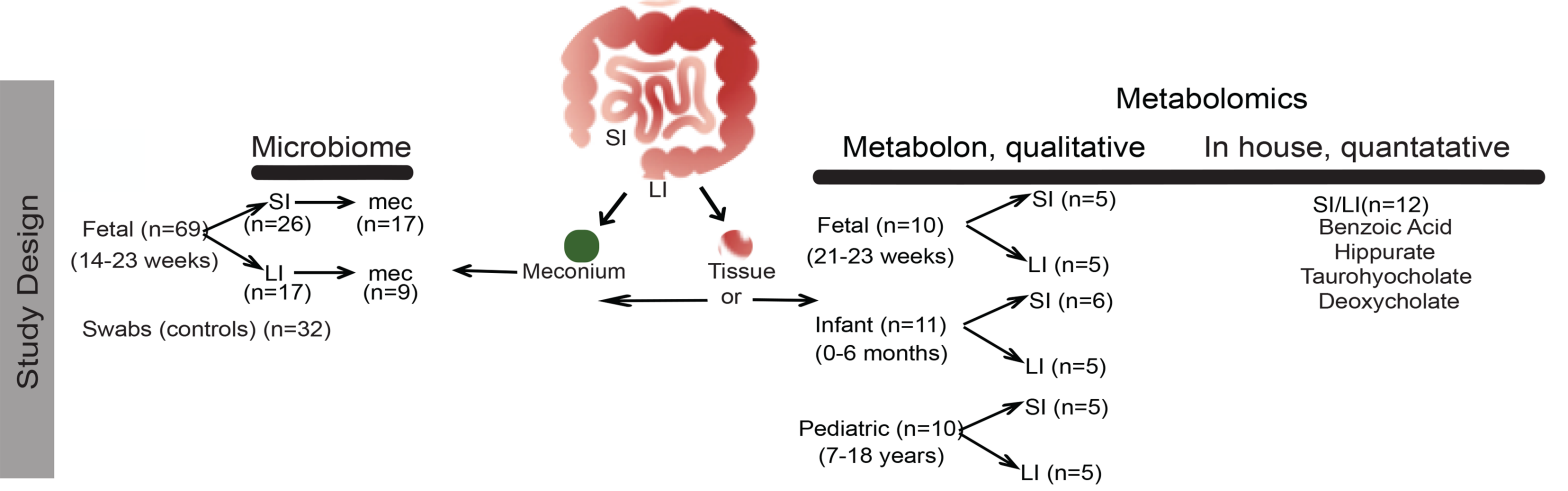
Study demographics. Workflow of sample cohort and delineation of microbiome or metabolomic analysis. Metadata and identification as either 16S or metabolomic analysis cohorts for all samples collected. SI, small intestine; LI, large intestine; Mec, meconium.

To assess in utero intestinal bacterial colonization, we attempted to amplify 16S rRNA sequences in 69 small (*n* = 26) and large (*n* = 17) intestinal samples and their respective meconium content (*n* = 17 and *n* = 9) from 14–23 weeks’ products of conception with no documented genetic abnormalities (Table 1). Additionally, we collected 32 swabs from the tissue procurement areas, hood, and tools as controls for environmental microbial contamination (Figure 1). In all 101 samples studied, the 16S rRNA gene sequencing did not produce a PCR product as shown in representative gel (Supplemental Figure 1, A and B; supplemental material available online with this article; https://doi.org/10.1172/jci.insight.138751DS1). As can be seen, these PCR reactions included positive and negative (kit reagents) controls that were appropriately amplified (positive) or not amplified (negative) (Supplemental Figure 1, A and B). One of the criticisms of the inability to amplify bacterial DNA from placentas has been the possibility of the placental tissue inhibiting the PCR reaction. Similar to inconsistent reports on the presence of a placental microbiome, data on placental inhibitory effects on the PCR amplification reaction have been conflicting: Onderonk and colleagues suggested that there is such an effect (22), whereas Kuperman et al., who spiked *E*. *coli* cultures to placental DNA and subsequently amplified *E*. *coli* DNA, suggested otherwise (11).

Given our inability to detect a prominent bacterial DNA component and thereby a robust intestinal microbiome, we alternatively explored microbial contributions in utero by investigating the presence of an in utero intestinal metabolome. To accomplish this, we performed global metabolomic analysis, including human and bacterially derived metabolites, using the Metabolon pipeline on 31 intestinal samples from 3 different age ranges: fetuses (21–23 weeks’ gestation) (Table 1); infants (0–6 months) (Table 2); and older children (pediatrics, 7–18 years) (Table 3 and Figure 1). Of the 10 fetal samples that underwent metabolomic analysis, 6 were matched to the ones that underwent microbial sequencing analysis (Table 1).

**Figure 2:**
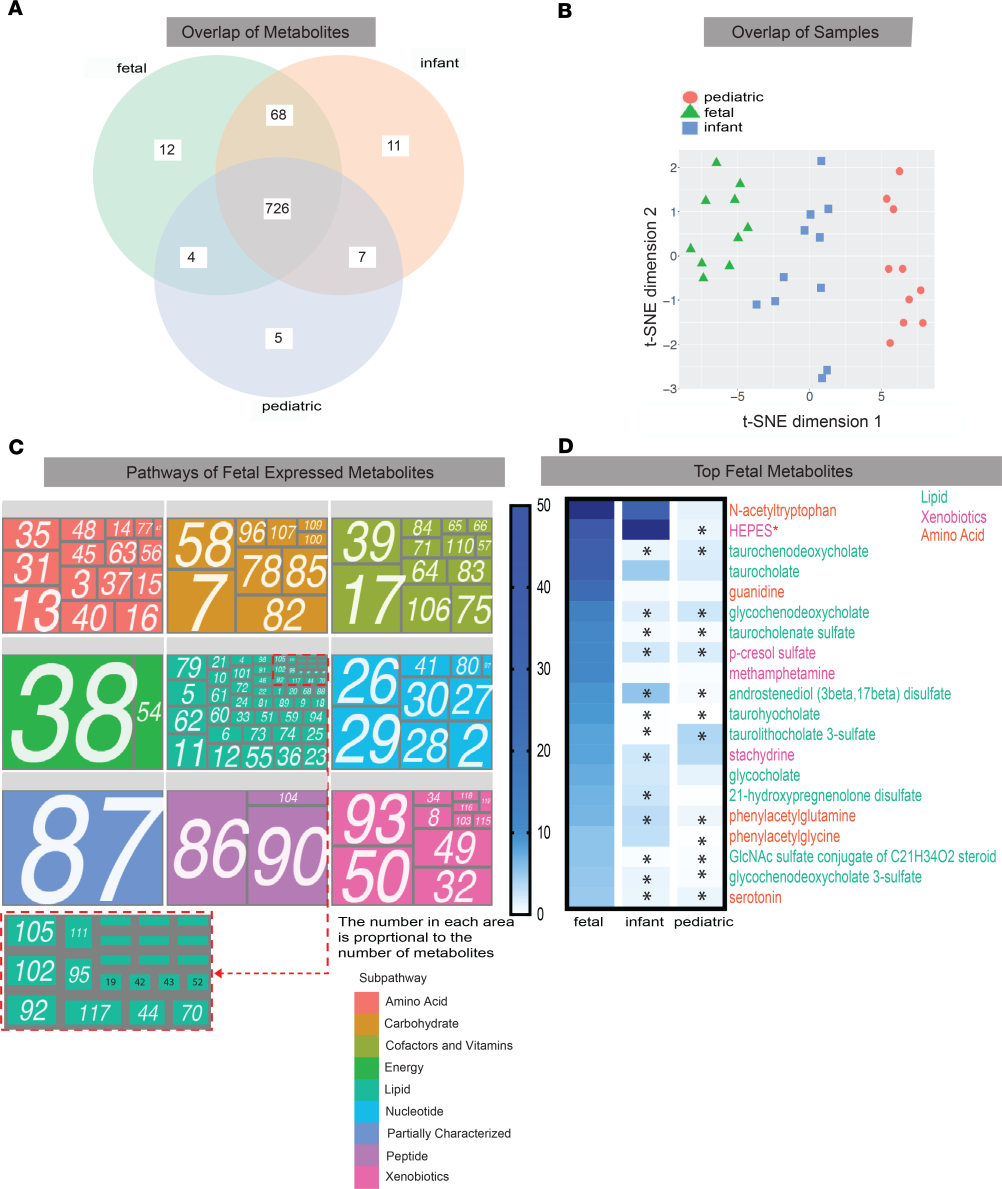
Description of metabolites expressed in fetal intestines. (**A**) Venn diagram of metabolites expressed both uniquely and shared between cohorts. (**B**) t-SNE analysis of bulk metabolomic profiles between cohorts. (**C**) Tree map depiction of metabolic pathways expressed in fetal intestine. Numbers correspond to pathways described in Supplemental Table 1. (**D**) Heatmap showing normalized expression of the top 20 fetal expressed metabolites. Labels color-coded to superpathways from tree maps in Figure 1C. Student’s 2-tailed *t* test with B-H correction for multiple comparison was used to compare expression values. *P < 0.05. Red asterisk indicates metabolite from media.

A total of 833 metabolites were identified (Figure 2A). Although most metabolites (726 of 833, 87%) were shared among all samples, each group also contained unique metabolites (fetal = 12, 1.4%; infant = 11, 1.3%; pediatric = 5, 0.6%) (Figure 2A). Furthermore, groups closer in age shared more metabolites than those with greater age difference. For example, fetal and infant samples shared 68 (8%) metabolites absent in pediatric samples, whereas pediatric and fetal samples only shared 4 (0.5%) absent from infants (Figure 2A). Of the 833 metabolites identified, 167 (20%) contained undetectable values in more than 50% of the samples. To determine if these missing values were due to expression levels below detection limits rather than random missingness, we classified metabolites into 4 groups with high (> 10), medium (5–9), low (1–4), and no missingness. We found that “missingness” correlated with overall lower expression (Supplemental Figure 1C). All 167 metabolites absent in more than 50% of the samples were filtered out, leaving 674 metabolites that were further imputed using previously published computational methods (23) and normalized by quantile normalization (Supplemental Figure 1D). t-SNE visualization of the 674 analyzed metabolites showed complete segregation of samples by age groups, suggesting that even though most metabolites were detected across all age groups, they were differentially abundant in the tissue based on age (Figure 2B).

Expressed metabolites were assigned to 119 subpathways (Supplemental Table 1) and classified into 9 superpathways (Figure 2C, Supplemental Figure 1E, and Supplemental Table 1). Abundance of metabolites in each subpathway within the superpathway was deduced for each age cohort (Figure 2C and Supplemental Figure 1E). Strikingly, metabolites in all subpathways were detected in fetal tissue (Figure 2C), including 11 xenobiotic subpathways (i.e., metabolites not naturally produced by humans). When assessing individual metabolites rather than broad pathways, the top 20 fetal expressed metabolites all belonged to 3 superpathways: amino acid, lipid, or xenobiotic. Although all were also present in pediatric and adult samples, they had differential abundances between fetal and infant or pediatric samples (Figure 2D). Similarly to Figure 2B, this suggests that the intestinal metabolome from 14–23 weeks’ gestation is distinct from the postnatal metabolome. Yet it is important to note we found that a couple of the high expression metabolites in fetal samples originated from the media the samples were processed in, such as HEPES. These media-derived metabolites are clearly notated throughout the figures (Figure 2D).

**Figure 3:**
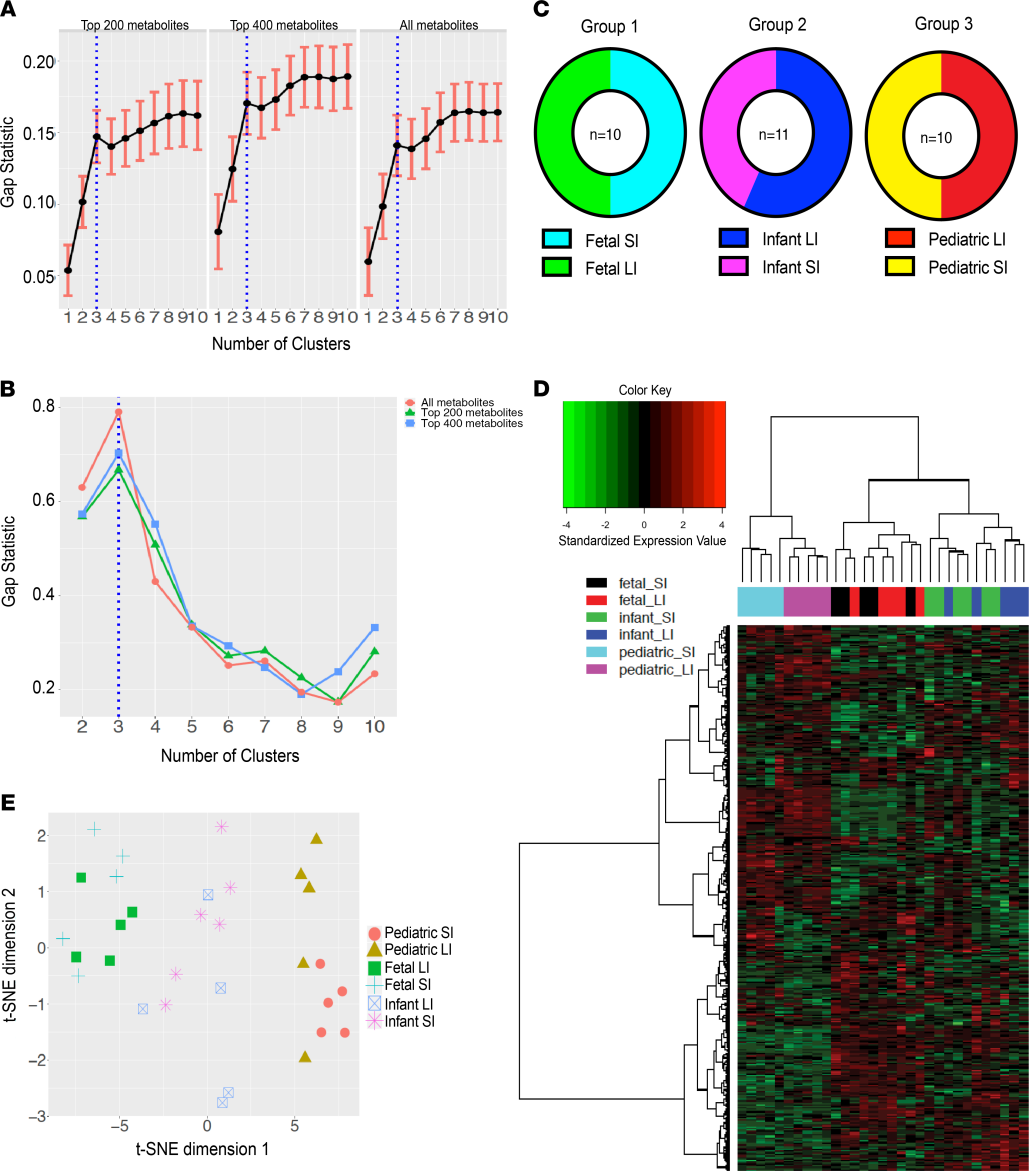
Metabolite clustering. (**A**) Gap statistics from 200, 400, and all most variable metabolites (determined by median absolute deviation). (**B**) Prediction strength for 200, 400, and all most variable metabolites. (**C**) K-means clustering was performed to generate 3 subgroups that were comprised entirely of age-matched intestinal tissue. (**D**) Hierarchical clustering with Ward linkage of samples using all metabolites. (**E**) t-SNE plot using all metabolites.

Next, we assessed whether unsupervised cluster analysis can separate the samples into pediatric, infant, and fetal groups. Gap statistics (24) using the 200 most variable metabolites resulted in 3 robust subgroups (Figure 3A). Varying the input to the 400th most variable or inputting all metabolites using Gap still supported 3 subgroups (Figure 3A). This was further validated by prediction strength algorithm (25) (Figure 3B) (highest at 3 clusters). The 3 clusters from K-means clustering (26) was comprised entirely of age-based intestinal tissue with Group 1 being fetal small intestine (SI) and large intestine (LI), Group 2 being infant SI and LI, and Group 3 being pediatric SI and LI (Figure 3C). Furthermore, using hierarchical clustering (HCL) (27) of all metabolites, we observed 3 clusters again (Figure 3D). Although fetal and infant samples did not further segregate with age group based on location (SI or LI), we observed that pediatric intestinal tissue could further subgroup into distinct SI and LI populations (Figure 3D). This clustering pattern may suggest that distinct SI and LI metabolomes are not set up until after infancy. t-SNE plot (Figure 3E) replicated HCL conclusions. In summary, cluster analysis of intestinal metabolites supported 3 metabolomic profiles specific to subject age.

**Figure 4:**
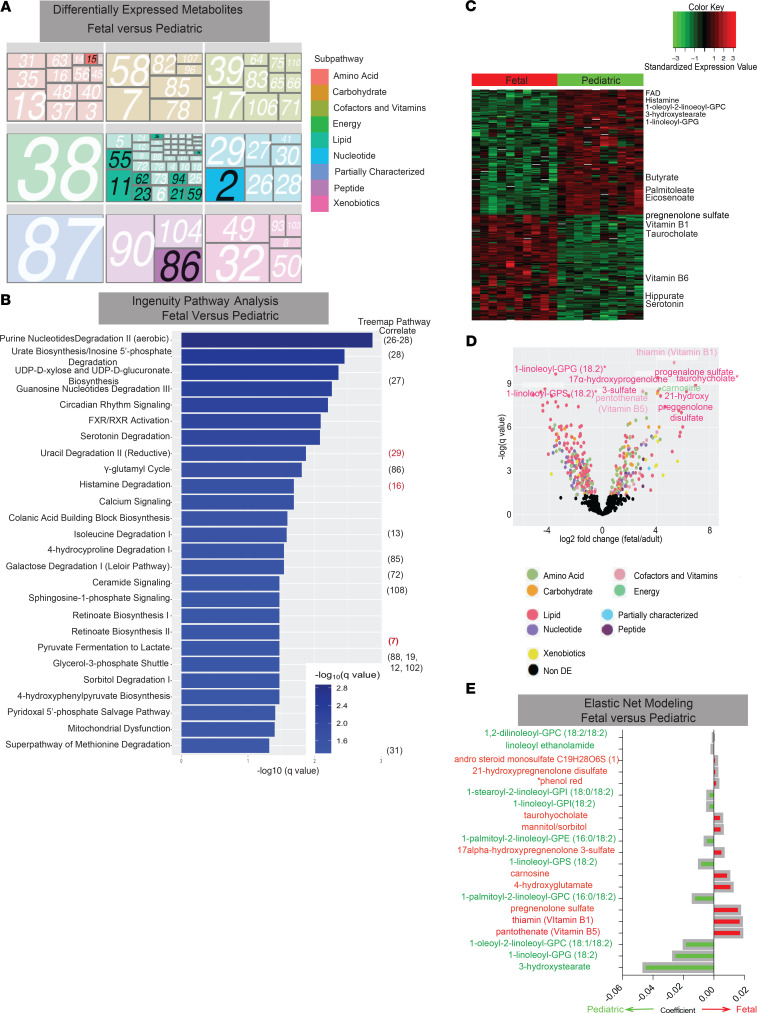
Differential expression of individual metabolites and metabolic pathways. (**A**) Tree map of differentially expressed subpathways between fetal and pediatric intestines. Numbers correspond to pathways described in Supplemental Table 1. Darker shades and black numbers indicate statistically significant differentially expressed pathways using Fisher’s exact test (*P* < 0.1). (**B**) Integrated pathway analysis for differentially expressed pathways between fetal and pediatric samples. Bar length is indicative of more significant *q* value. Numbers on right-hand side correlate to tree map pathways in Supplemental Table 1. (**C**) Heatmap of differential expression of individual metabolites. Each column represents one sample and each row represents one metabolite. For a complete list of metabolites, see Supplemental Table 5. (**D**) Volcano plot of differentially expressed metabolites between fetal and pediatric samples. Metabolites with positive *x* axis are those with higher expression in fetus. The 8 most significant metabolites are labeled with the metabolite name. All significant differentially expressed metabolites (*q* < 0.05) are color-coded by superpathway from **A**. (**E**) Top 20 metabolites from elastic net model. Red asterisk indicates metabolite from media.

After confirming metabolomic profiles were age-specific, we analyzed which subpathways were differentially expressed between the cohorts. Multiple subpathways comprised of individual metabolites were differentially present in fetal, infant or pediatric samples (Figure 4A, Supplemental Figure 2A, and Supplemental Table 2). Illustrated by darker shades/bold color on the subpathway tree plots, there were 12 differentially expressed subpathways falling into 4 superpathways between fetal and pediatric samples (Figure 4A), 12 subpathways in 2 superpathways for fetal and infant, and 11 subpathways within 6 superpathways for infant and pediatric groups with *P* values less than 0.1 (Supplemental Figure 2A). Of interest, 2 xenobiotic subpathways — xanthine metabolism (no. 8) and analgesics/anesthetics (no. 93) — were differentially present in the fetal versus infant comparisons but similar in the fetal versus pediatric comparisons (Figure 4A and Supplemental Figure 2A). Both xanthine and analgesics/anesthetics are xenobiotics that have a higher exposure prevalence in adulthood than in infants, and thus their presence in fetal samples may reflect maternal metabolites reaching the fetal intestines in utero.

Furthermore, we used Ingenuity Pathway Analysis (IPA) (28) (Figure 4B, Supplemental Figure 2, B and C, and Supplemental Table 3), which served as an independent bioinformatic validation. IPA identified physiologic pathways that were significantly altered in fetal samples compared with pediatric samples, including circadian rhythm signaling and sorbitol metabolism, both of which are critical to the maintenance of intestinal motility (Figure 4B and Supplemental Table 3) (29, 30). We hypothesize these pathways were upregulated in pediatric samples compared with fetal samples because they support the notion that intestinal motility is absent before the gestational age of 16–18 weeks and does not fully develop until closer to full gestation (31).

To investigate age-specific individual metabolite signatures, we next compared metabolite enrichment between the groups (fetal vs. pediatric = 357, 53%; fetal vs. infant = 181, 27%; and infant vs. pediatric = 251, 37%) (Figure 4C, Supplemental Figure 3A, and Supplemental Tables 4–6). In confirmation of earlier findings (Figure 2), there was little variation between individuals within a cohort and complete segregation of metabolite expression between cohorts, i.e., individual fetuses displayed similar expression to each other and distinct expression from pediatric samples (Figure 4C and Supplemental Figure 3A). Interestingly, 9 of the 10 most differentially expressed metabolites between fetal and pediatric samples were lipids and vitamins, with the majority of these enriched in the fetus (Figure 4D). In contrast, when fetal metabolite expression was compared with infant, there was differential expression of metabolites belonging to lipid, amino acid, and xenobiotic superpathways (Supplemental Figure 3B).

Using the expression of individual metabolites, predictive modeling with elastic net was able to segregate individual samples into the correct cohorts with perfect accuracy (AUC = 1) for fetal versus pediatric and infant versus pediatric comparisons and near perfect accuracy (AUC = 0.99) for fetal versus infant comparisons (Figure 4E and Supplemental Figure 3, C and D). Specifically, when analyzing the top 20 metabolites contributing to the elastic net model, 3-hydroxystearate, 1-linoleoyl-GPG (18:2), and 1-oleoyl-2-linoleoyl-GPC (18:1/18:2) were the top 3 contributors and preferentially enriched in pediatric samples as indicated by a negative coefficient (Figure 4E and Supplemental Table 7). All 3 metabolites are involved in fatty acid metabolism used by both host cells and commensals (32). The elevation of fatty acid metabolism in the pediatric cohort aligns with the well-established abundant microbiota present in pediatric intestines. Furthermore, 2 progesterone derivatives, 21-hydroxypregnenolone disulfate and progenolone sulfate, were enriched in fetal samples that significantly contributed to the model (Figure 4E and Supplemental Table 7). Furthermore, using the elastic net model, there were 59 metabolites with AUC values of 1.00 in the fetal versus pediatric model, indicating that these 59 metabolites could be used to segregate the samples to the correct cohort with perfect accuracy (Supplemental Table 7).

In the fetal versus infant model, there were 2 individual metabolites with 1.00 AUC values: cortisone, a glucocorticoid steroid previously shown to increase in the murine small intestine after delivery (33), enriched in the infant, and taurocholate, a primary bile acid, enriched in the fetus (Supplemental Figure 3C and Supplemental Table 7). Furthermore, the top 2 metabolites with the greatest contribution to the model, N-acetylthreonine and 2-hydroxybutyrate/2-hydroxyisobutyrate (Supplemental Figure 3C and Supplemental Table 7), were enriched in the infant intestines. N-acetylthreonine is important for mucin production, and deficiencies have been reported in formula-fed infants and subsequently associated with necrotizing enterocolitis a neonate-specific morbidity (34). Increased abundance of 2-hydroxybutyrate/2-hydroxyisobutyrate is indicative of oxidative stress, and, as such, low levels in a fetus may be critical because high oxidative stress has been associated with adverse birth outcomes and improper fetal programming (35). When comparing infant with pediatric samples, there were 12 metabolites with perfect 1.00 AUC values (Supplemental Table 7). Collectively, each elastic net model and the evaluation of multiple metabolites with perfect AUC values across models support our hypothesis that intestinal metabolomes are age-specific throughout prenatal to late pediatric time periods.

**Figure 5:**
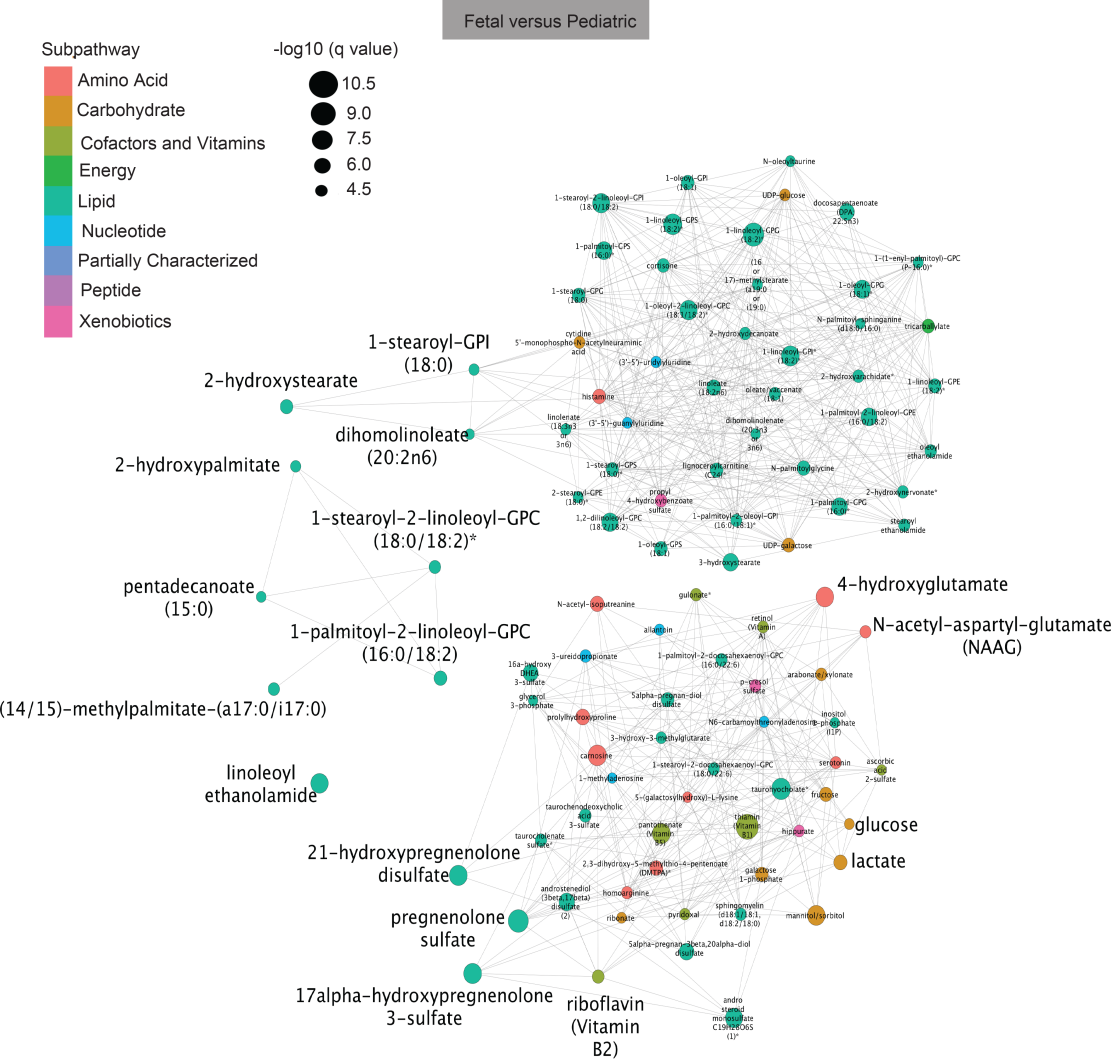
Network analysis of fetal versus pediatric metabolites. Tanimoto network analysis for fetal versus pediatric individual metabolites. Size of circle reflective of *q* value, color of circle reflective of superpathway.

Given the difficulty to map many metabolites into subpathways and superpathways, we additionally used Tanimoto networks (23, 36) to determine the relationship between the expressed metabolite (Figure 5 and Supplemental Figure 4, A and B). In these plots, the size of the circle directly correlates to the *q* value in the comparison between fetal and pediatric (Figure 5), fetal and infant (Supplemental Figure 4A), and infant and pediatric samples (Supplemental Figure 4B). The fewer connections a metabolite has, the more different its expression is between the 2 groups. In conformation of our elastic net modeling, we identified multiple of the same differentially expressed metabolites between fetal and pediatric samples, including progenolone sulfate, linoleoyl ethanolamide, and 21-hydroxypregnenolone disulfide (Figure 4E and Figure 5).

**Figure 6:**
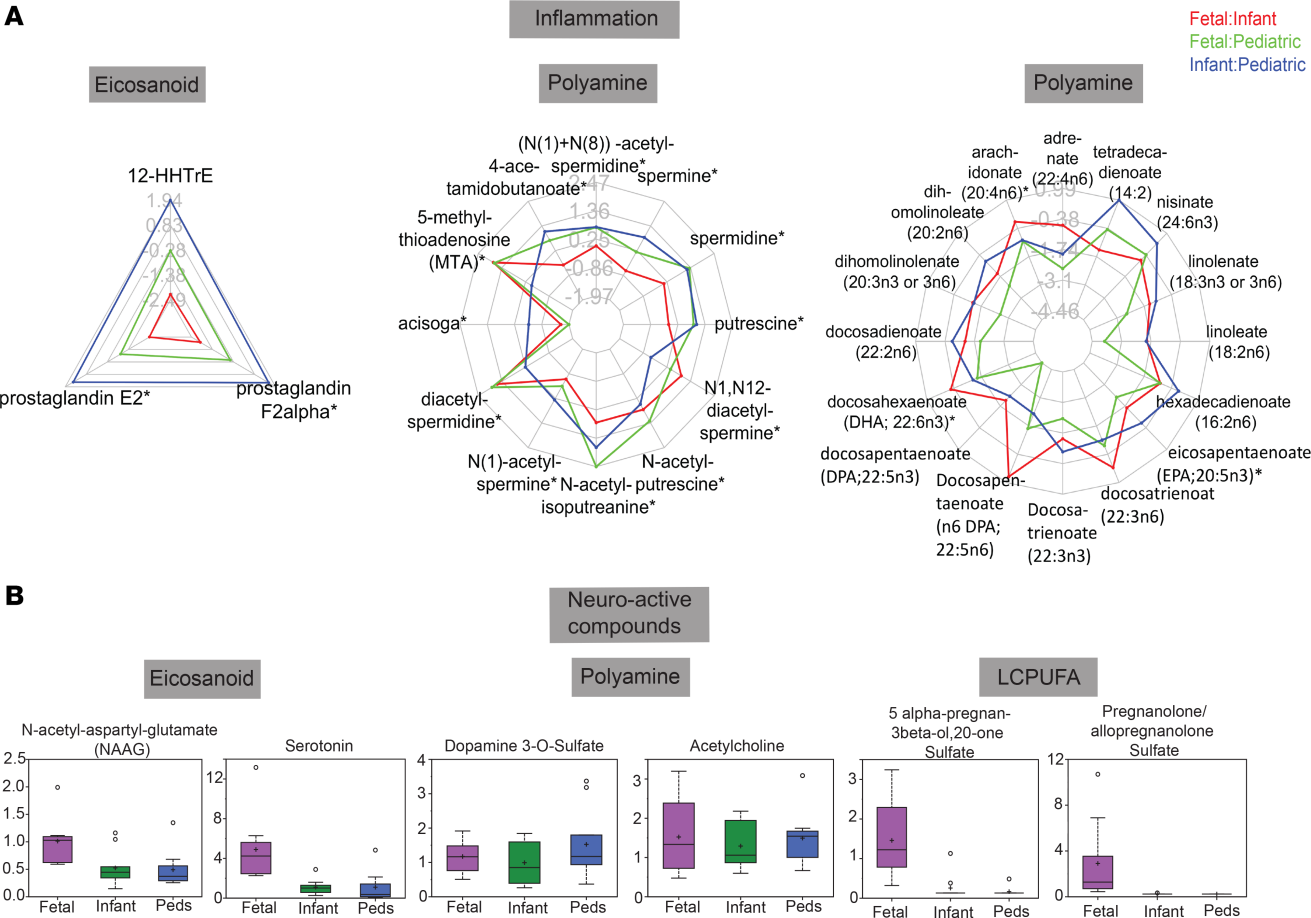
Inflammatory and neuroactive metabolic signatures. (**A**) Radar plots of metabolites implicated in inflammation. (**B**) Box-and-whisker plots of individual metabolite expression for neuroactive metabolites. *P < 0.05.

Next, we specifically analyzed metabolite signatures reflective of intestinal function and observed differential expression of some regulators of inflammation (Figure 6A) and neuroactive metabolites (Figure 6B) in fetuses, confirming that fetal intestines are active across multiple physiological pathways. For example, 5-methylthioadenosine (37) and eicosapentaenoate (38), 2 potent antiinflammatory molecules, were enriched in the fetuses (Figure 6A), whereas potent inflammatory mediators, such as prostaglandin E2, were reduced in the fetuses (Figure 6A). Examples of neuroactive compounds enriched in the fetus were NAAG, serotonin, 5α-pregnan-3β-ol-20-one sulfate, and allopregnanolone sulfate, whereas dopamine and acetylcholine were present in similar amounts across all 3 groups (Figure 6B).

**Figure 7:**
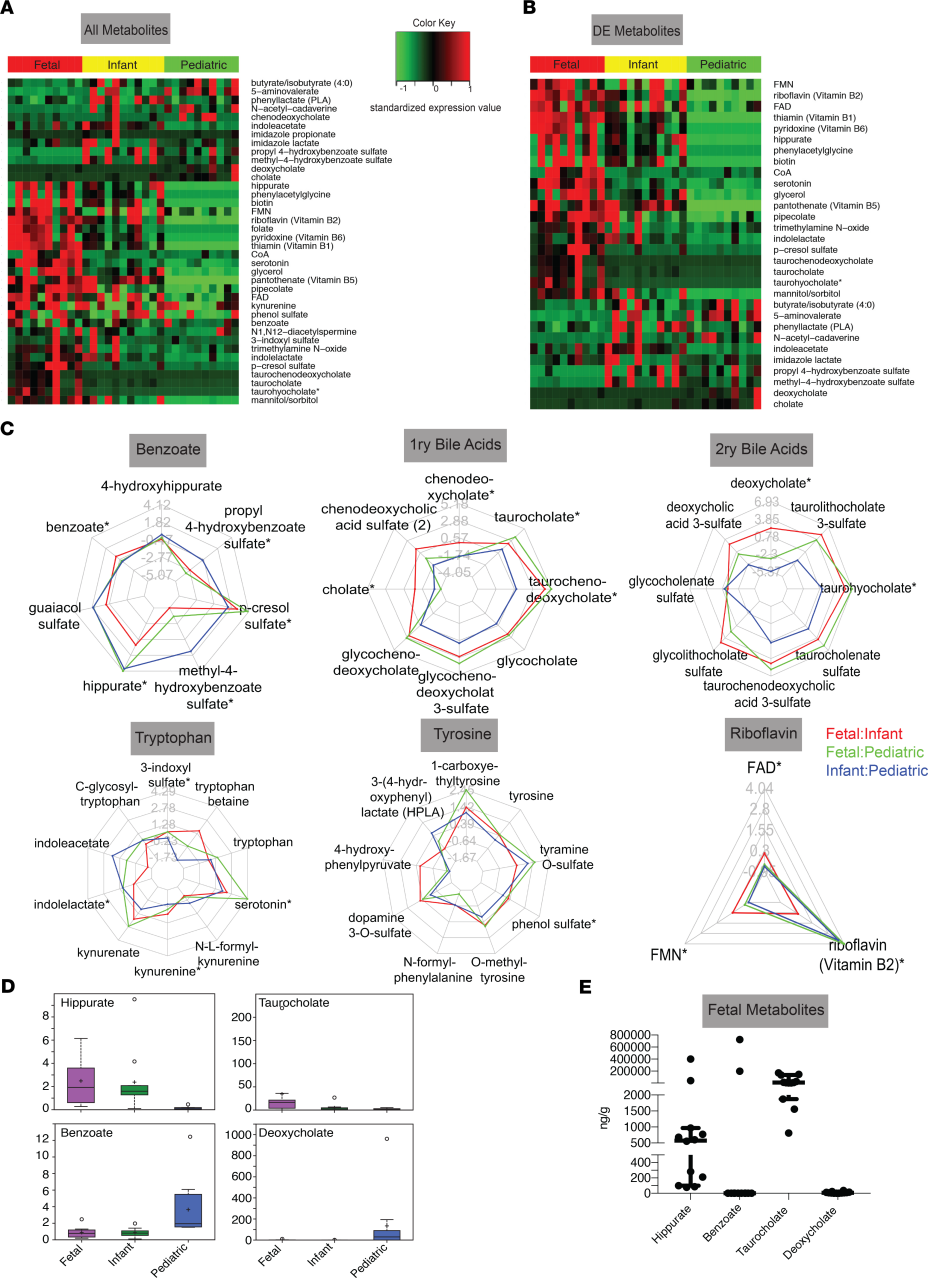
Microbial metabolite signatures in fetal, infant, and pediatric intestines. (**A**) Relative expression of all metabolites produced by eithermicrobes or the host in response to microbial presence identified from a literature search in each sample set. (**B**) Differentially expressed microbial-associated metabolites. (**C**) Radar plots for expression of microbial-associated metabolites in 6 families. (**D**) Box-and-whisker plots of relativevalues of microbial metabolites. (**E**) Quantitative values of fetal microbial-associated metabolites. *P < 0.05.

Though we failed to detect microbial 16S rRNA (Supplemental Figure 1, A and B), we hypothesized that microbial byproducts may still be present in the intestine in utero. Metabolites were classified as microbial if they could be linked to bacterial metabolism through a literature search. Of the 674 filtered metabolites, 38 (5.6%) represented metabolites that could be linked to the microbiome, produced by either microbes or the host in response to microbes (Figure 7A, Supplemental Table 8, and Supplemental Table 9). Of the 38 microbial-associated metabolites, 30 were identified as differentially expressed between the 3 cohorts (Figure 7B and Supplemental Table 8). These 30 metabolites belonged to 8 metabolic families, depicted in radar plots (Figure 7C): tyrosine, tryptophan, primary, secondary bile acids, riboflavin, pentothenate, thiamine, and benzoate. Pentothenate and thiamine derivatives were not pictured due to 2 or fewer metabolites per group and thus cannot be represented in radar plots. Strikingly, almost all metabolites were present to some extent in the fetuses and many microbial metabolites were enriched in fetuses compared with both infant and pediatric samples (Figure 7, A and B, and Supplemental Table 8). Such fetal-enriched metabolites are as follows: benzoate metabolites p-cresol sulfate and hippurate; bacterially linked metabolites associated with higher Shannon diversity (39); 3 bile acids, taurohyocholate, taurochenodeoxycholate, and taurocholate; and one tryptophan metabolite, serotonin (Figure 7C). Additionally, we report multiple microbial metabolites enriched in early development (i.e., fetal/infant compared with pediatric), including riboflavin (vitamin B2), indolelactate, and indoleacetate (Figure 7C). To validate whether some of these metabolites were indeed differentially present in the fetal intestinal tissues, we performed quantitative analysis of 4 commercially available metabolites with differential expression (Figure 7D). Quantitative analysis of 12 fetal SI/LI samples (Table 1) confirmed the abundance of patterns observed with the Metabolon pipeline: hippurate and taurocholate were present in majority of the fetal samples, whereas benzoate and deoxycholate were largely undetectable (Figure 7E). In summation, using metabolomic profiling we have identified a continuum of distinct intestinal metabolomes from gestational to late pediatric age. Last, we report that multiple metabolites associated with microbiota are present in fetuses.

## Discussion

Newborn infants are more susceptible to sepsis than any other age group (1). Though newborns are exposed to large numbers of microbes during and shortly after birth, most newborns are born healthy and do not suffer from neonatal infections. It has long been believed that the neonatal immune system is immature and not fully functional, derived from evidence such as naive B and T cell dominance in cord blood (40) and neonates’ poor vaccine responses (41), among others. However, recent evidence suggests that the neonatal immune system is well developed but, in some contexts, serves a different function from that of adults. Examples of such work are the education and maturation of fetal T regulatory cells to noninherited maternal antigens in utero (42); the detection of novel fetal/neonatal suppressive cell types that prevent T cell activation in neonatal circulation (43, 44); and the presence of phenotypically mature B and T cells in fetal organs (2–4, 45). As such, it is likely that the neonatal immune system is at different stages of maturation, depending on the cell type and location, with certain aspects that are immature, attributing to, for example, neonates’ poor vaccine response, whereas other parts of the early-life immunity are fully developed with either canonical or novel functions.

To begin unraveling in utero immune function, we were interested in identifying potential antigens that fetal intestinal T cells may recognize (2–4, 43). It is reported that fetal T cells specific to malaria antigens are generated during gestation and confer protection for the infant later in life (20). With this in mind, we attempted to detect microbiota that could potentially be recognized by fetal T cells in the fetal small/large intestines and meconium. We were unable to detect any microbial signatures in the fetal tissue or meconium samples, congruent with work from multiple other groups who were also unable to detect a microbiota from gestational tissues (7–11). However, recently Rackaityte and colleagues reported bacterial presence via scanning electron microscopy and limited signatures by 16S rRNA sequencing in a similar sample set (12). The discrepancy between our inability to amplify bacterial DNA and the findings by Rackaityte and colleagues exemplifies the debate over the existence of an in utero microbiome, with some studies identifying a low biomass microbiome (13–18) and others finding no microbial signatures beyond background levels in animals and humans (7–11, 46–48). The controversy about the existence of a placental and fetal microbiome cannot be resolved solely with the data presented in this manuscript. We propose that a potential resolution respecting previous evidence presented on both sides of the argument would examine larger cohorts of pregnancy-matched fetal organs such as the intestine, the placenta sampled from multiple locations, including the decidua, and both the placental villi and maternal blood from the intervillous space and maternal circulation. Sampling should be done with strict guidelines and standard operating procedures with multiple experts examining the results (49).

Though we did not detect any bacterial DNA, we hypothesized that microbial byproducts may be present in fetal intestines in utero–derived from either the maternal microbiota or a low biomass fetal microbiome. Using the metabolomic analysis pipeline from Metabolon and multiple computational analyses, we have provided a comprehensive report of the in utero human intestinal metabolome. Our data show that metabolomic profile of human intestines is changing throughout prenatal to late teenage time points, and that within each developmental stage, fetal-, infant-, and pediatric-specific metabolomic signatures are present. In fetal, infant, and pediatric intestines, we detected metabolites in all 9 superpathways analyzed (amino acid, carbohydrate, cofactors, vitamins, energy, lipid, nucleotide, peptide, partially characterized molecules, and xenobiotics). However, the subpathways within the superpathways highly varied between our groups. Focusing on the fetal intestines, the top 20 most expressed metabolites belonged to lipid, xenobiotic, and amino acid pathways. Furthermore, of these metabolites, 15 of 20 were differentially expressed in infant and/or pediatric intestines, highlighting developmentally specific metabolomes. Using multiple modeling and comparative algorithms we were further able to identify in utero–enriched metabolites and metabolic pathways, including multiple inflammatory and neuroactive compounds.

As previously stated in our fetal samples, we detected many xenobiotic products. This was particularly interesting because xenobiotic products in after-birth intestine are predominately those ingested, including food components, environmental chemicals, and pharmaceuticals, or are those produced by the gut microbiome (50). A fetus begins swallowing amniotic fluid at 10–14 weeks’ gestation (51). This coupled with the detection of certain xenobiotics in the fetus (i.e., plant components) suggests that some food metabolites ingested by the mother are reaching the fetus. One example of a commonly ingested substance with metabolites reaching the fetus was the detection of B vitamins. Specifically, vitamins B1 and B5 were 2 fetal-abundant metabolites that strongly contributed to our elastic net model, which accurately predicted segregation of fetal and pediatric metabolomes based on individual metabolite signatures. Both vitamin B1 and vitamin B5 are common components of prenatal vitamin mixtures and likely represent maternal transfer of vitamins reaching the fetal compartment secondary to increased maternal consumption (52).

Because we failed to amplify microbial sequences in our efforts, and knowing that if an in utero microbiome does exist, it persists at low biomass (13–18), we hypothesize that the majority of microbial metabolites detected in the fetal cohort represent vertically transmitted metabolites produced by or in response to the maternal microbiota. This position is supported by reports that neonatal mice born to mothers with a microbiota have increased xenobiotic metabolic signatures compared with germ-free pups (19). However, it is also possible that some microbial metabolites could potentially be derived from or in response to microbes in the fetal intestine. Further studies evaluating matched fetal and maternal samples are needed to differentiate between these possibilities.

Diving deeper into these microbial metabolites we identified one benzoate metabolite, p-cresol sulfate, enriched in fetal samples compared with infant and pediatric samples. p-Cresol is generated by bacterial fermentation of tyrosine and phenylalanine and is further conjugated to p-cresol sulfate in the liver and by intestinal microbes (53). Moreover, alterations in fecal p-cresol levels have been associated with microbiome disturbances in IgA pathologies (54). We speculate that high in utero levels of intestinal p-cresol may be a preemptive measure to prepare to tolerate the colonizing gut microbiome and IgA delivered in breast milk. Congruent with this thinking that healthy microbiome signatures would be enriched during gestation in preparation for early life colonization, hippurate was enriched in both fetal and infant samples compared with pediatric ones. Hippurate metabolism is the result of microbial transformation of food and host-derived metabolites and has been associated with increased Shannon diversity seen in healthy microbiomes (39). Our analysis identified a number of primary and secondary bile acids enriched in fetal groups compared with postnatal groups: taurohyocholate, taurochenodeoxycholate, and taurocholate. Primary bile acids are produced in the liver and then absorbed by the small intestine. Upon crossing the intestinal lumen, microbiota dehydroxylate/deconjugate bile acids result in secondary bile acids such as taurohyocholate (55). Interestingly, intestinal bile acids have recently been shown to alter abundance and type of mucosal regulatory T cell (56), suggesting that the differential presence of various bile acids across the 3 age groups may be important in establishing/regulating mucosal immunity.

In addition to postulated microbiota-produced metabolites, we also identified host-made metabolites connected with the intestinal microbiome. In multiple computational analyses, progesterone metabolites were identified as differentially expressed in fetal samples compared with infant and pediatric samples. An elevation in progesterone metabolites in the fetal samples is likely due to its synthesis by the tissues at the fetal–maternal interface (57). However, the detection of the progesterone metabolites in the fetal intestines is consistent with recent findings, in which one of the few bacterial species isolated from the fetal intestines grew preferentially in cultures containing pregnancy levels of progesterone in carbon-limited environments (12).

Serotonin, a tryptophan metabolite, was also differentially expressed in fetal versus pediatric and adult tissue. Serotonin is produced within the enterochromaffin cells in response to direct microbial interactions (58). Serotonin’s elevation within the fetal intestine, where there is likely limited live microbial contact, suggests that contact with microbial components rather than live microbes could be sufficient for enterochromaffin cell metabolomic responses. However, it is also possible that serotonin is maternally derived and preferentially transported across the placenta, or that serotonin is derived in the placenta from maternal tryptophan precursors (59). Furthermore, many tryptophan metabolites such as 3-indoxyl sulfate (60), which was enriched in the fetal intestine, represent an intriguing link between the microbiome and intestinal immunity, through their activity as AHR ligands. AHR ligands commonly induce IL-22 production and regulate immune development (21). Congruent with this finding, ILC3s, a major producer of IL-22, have recently been reported to be enriched in fetal intestines compared with infant samples (2). Taken together, these findings may represent a means by which tryptophan metabolites produced by the maternal microbiome or a low biomass fetal microbiome signal through AHRs to mediate IL-22 production by ILC3s and other immune cells, which subsequently prepare the fetal intestine for postnatal microbial colonization.

The main limitations of our study were the relatively small sample size of each cohort. Furthermore, the fetal samples represent only 1 month of gestation. It would be interesting to additionally assess the metabolome of first and third trimester intestines to evaluate intestinal metabolomic changes throughout gestational development. However, it is ethically and technically challenging to obtain tissue from either of these time points. Though we reported a complex fetal metabolome, we were unable to determine if metabolites were fetal-derived or maternally derived. It would be valuable for future studies to compare in utero intestinal metabolites to matched placental and decidual (maternal uterine lining) samples from the same product of conception to determine if metabolites, particularly those associated with the microbiota, were of fetal origin or maternally derived that crossed the placenta. Due to limitations of tissue collection, we were unable to generate this cohort.

Because we were unable to detect robust microbial 16S rRNA gene sequences, we propose that microbial byproducts from the maternal microbiome either are vertically transmitted to the fetus during pregnancy or originate from a low biomass fetal microbiome that our techniques could not detect. In either case, microbial byproducts identified in this study potentially prime the fetal immune system for postnatal microbial encounters during and after birth. Malfunctions in this priming may thus result in uncontrolled inflammation in newborns and subsequently lead to early onset neonatal sepsis. Collectively, this report of the fetal intestinal metabolome identified many distinct in utero signatures that should be explored in future studies to investigate human intestinal development and function during gestation.

## Methods

### Sample collection

Fetal intestine was obtained from the University of Pittsburgh Biospecimen Core from electively terminated products of conception (14–23 weeks’ gestation). Products of conception were collected from dilation and evacuation procedures with nonpharmacological, mechanical dilation via Dilapan-S. No fetal subjects had reported genetic abnormalities. In respect of patient confidentiality and safety, limited clinical information was collected for fetal samples. All demographic information that could be legally and respectfully obtained is shown in Table 1. Infant samples were collected after intestinal resections for the presumed diagnosis listed in Table 1. After receiving fetal and infant samples, meconium was removed, and a small piece was cut with a sterile blade and immediately snap-frozen and stored at –80°C until shipment to Metabolon. Pediatric ileal and colonic biopsies were collected from patients undergoing colonoscopy for evaluation of abdominal pain, diarrhea, and/or bloody stools (Table 3). Biopsies were immediately placed in liquid nitrogen after collection and then transferred to –80°C, until processing.

### 16S microbiome analysis

DNA was extracted from 101 samples using the PureLink Microbiome DNA Purification Kit (Invitrogen) according to the manufacturer’s instructions and following a 2-minute bead-beating step (Biospec). The V4 region of the bacterial 16S rRNA gene was amplified from the extracted DNA using the 515F and 806R barcoded primers as previously described (36). Each PCR reaction consisted of 2 μL 515F primer (10 μM), 2 μL 806R primer (10 μM), 25 μL PrimeSTAR Max PCR mix (Takara), 17 μL ultrapure water, and 10 μL of sample DNA. DNA amplification consisted of an initial denaturing step for 3 minutes at 95°C followed by 35 cycles of denaturation (98°C for 10 seconds), annealing (55°C for 5 seconds), and extension (72°C for 20 seconds) and with a final elongation step at 72°C (for 1 minute). PCR products were viewed on a 1% agarose gel.

### Metabolomic data acquisition

#### Sample accessioning.

After receipt, samples were inventoried and immediately stored at –80°C. Each sample was accessioned into the Metabolon LIMS system and was assigned a unique identifier by the LIMS that was associated with the original source identifier only. This identifier was used to track all sample handling, tasks, results, etc. The samples (and all derived aliquots) were tracked by the LIMS system. All portions of any sample were automatically assigned their own unique identifiers by the LIMS when a new task was created; the relationship of these samples was also tracked.

#### Sample preparation.

Samples were prepared using the automated MicroLab STAR system from Hamilton Company. Several recovery standards were added before the first step in the extraction process for quality control (QC) purposes. To remove protein, small molecules bound to protein or trapped in the precipitated protein matrix, and to recover chemically diverse metabolites, proteins were precipitated with methanol under vigorous shaking for 2 minutes (Glen Mills GenoGrinder 2000) followed by centrifugation. The resulting extract was divided into 5 fractions: 2 for analysis by 2 separate reverse phase (RP)/ultrahigh-performance liquid chromatography tandem mass spectroscopy (UPLC-MS/MS) methods with positive ion mode electrospray ionization (ESI); 1 for analysis by RP/UPLC-MS/MS with negative ion mode ESI; 1 for analysis by HILIC/UPLC-MS/MS with negative ion mode ESI; and 1 sample was reserved for backup. Samples were placed briefly on a TurboVap (Zymark) to remove the organic solvent. The sample extracts were stored overnight under nitrogen before preparation for analysis.

#### Metabolon workflow.

All samples were analyzed by Metabolon with their standard pipeline. Raw data were filtered and normalized by Metabolon’s proprietary pipeline.

#### Quality assurance/QC.

Several types of controls were analyzed in concert with the experimental samples: a pooled matrix sample, generated by taking a small volume of each experimental sample (or, alternatively, use of a pool of well-characterized human plasma), served as a technical replicate throughout the data set; extracted water samples served as process blanks; and a cocktail of QC standards that were carefully chosen not to interfere with the measurement of endogenous compounds and spiked into every analyzed sample, allowed instrument performance monitoring, and aided chromatographic alignment (Supplemental Figure 5, A and B). Instrument variability was determined by calculating the median relative SD (RSD) for the standards that were added to each sample before injection into the mass spectrometers. Overall process variability was determined by calculating the median RSD for all endogenous metabolites (i.e., noninstrument standards) present in 100% of the pooled matrix samples. Experimental samples were randomized across the platform run with QC samples spaced evenly among the injections, as outlined in Supplemental Figure 5C.

#### UPLC-MS/MS.

All methods used a Waters ACQUITY UPLC and a Thermo Scientific Q-Exactive high-resolution accurate-mass spectrometer interfaced with a Heated Electrospray Ionization (HESI-II) source and Orbitrap mass analyzer operated at 35,000 mass resolution. The sample extract was dried and then reconstituted in solvents compatible to each of the 4 methods. Each reconstitution solvent contained a series of standards at fixed concentrations to ensure injection and chromatographic consistency. One aliquot was analyzed using acidic positive ion conditions, chromatographically optimized for more hydrophilic compounds. In this method, the extract was gradient eluted from a C18 column (Waters UPLC BEH C18-2.1 × 100 mM, 1.7 μm) using water and methanol, containing 0.05% perfluoropentanoic acid (PFPA) and 0.1% formic acid (FA). Another aliquot was also analyzed using acidic positive ion conditions; however, it was chromatographically optimized for more hydrophobic compounds. In this method, the extract was gradient eluted from the same aforementioned C18 column using methanol, acetonitrile, water, 0.05% PFPA, and 0.01% FA and was operated at an overall higher organic content. Another aliquot was analyzed using basic negative ion optimized conditions using a separate dedicated C18 column. The basic extracts were gradient eluted from the column using methanol and water but with 6.5 mM ammonium bicarbonate at pH 8. The fourth aliquot was analyzed via negative ionization after elution from a HILIC column (Waters UPLC BEH Amide 2.1 × 150 mM, 1.7 μm) using a gradient consisting of water and acetonitrile with 10 mM ammonium formate, pH 10.8. The MS analysis alternated between MS and data-dependent MS^n^ scans using dynamic exclusion. The scan range varied slighted between methods but covered 70–1000 *m/z*. Raw data files are archived and extracted as described below.

#### Bioinformatics.

The informatics system consisted of the following 4 major components: the Laboratory Information Management System (LIMS), the data extraction and peak identification software, data processing tools for QC and compound identification, and a collection of information interpretation and visualization tools for use by data analysts. The hardware and software foundations for these informatics components were the LAN backbone and a database server running Oracle 10.2.0.1 Enterprise Edition.

#### LIMS.

The purpose of the Metabolon LIMS system was to enable fully auditable laboratory automation through a secure, easy-to-use, and highly specialized system. The scope of the Metabolon LIMS system encompasses sample accessioning, sample preparation, instrumental analysis, reporting, and advanced data analysis. All of the subsequent software systems are grounded in the LIMS data structures. It has been modified to leverage and interface with the in-house information extraction and data visualization systems as well as third-party instrumentation and data analysis software.

#### Data extraction and compound identification.

Raw data were extracted, peak-identified, and QC-processed using Metabolon’s hardware and software. These systems are built on a web-service platform using Microsoft’s.NET technologies, which run on high-performance application servers and fiber channel storage arrays in clusters to provide active fail-over and load balancing. Compounds were identified by comparison to library entries of purified standards or recurrent unknown entities. Metabolon maintains a library based on authenticated standards that contain the retention time/index (RI), mass-to-charge ratio (*m/z)*, and chromatographic data (including MS/MS spectral data) on all molecules present in the library. Furthermore, biochemical identifications are based on 3 criteria: retention index within a narrow RI window of the proposed identification, accurate mass match to the library plus or minus 10 ppm, and the MS/MS forward and reverse scores between the experimental data and authentic standards. The MS/MS scores are based on a comparison of the ions present in the experimental spectrum with the ions present in the library spectrum. Although there may be similarities between these molecules based on one of these factors, the use of all 3 data points can be used to distinguish and differentiate biochemicals. More than 3300 commercially available purified standard compounds have been acquired and registered into LIMS for analysis on all platforms for determination of their analytical characteristics. Additional mass spectral entries have been created for structurally unnamed biochemicals, which have been identified by virtue of their recurrent nature (both chromatographic and mass spectral). These compounds have the potential to be identified by future acquisition of a matching purified standard or by classical structural analysis.

#### Curation.

A variety of curation procedures were carried out to ensure that a high-quality data set was made available for statistical analysis and data interpretation. The QC and curation processes were designed to ensure accurate and consistent identification of true chemical entities and to remove those representing system artifacts, mis-assignments, and background noise. Metabolon data analysts use proprietary visualization and interpretation software to confirm the consistency of peak identification among the various samples. Library matches for each compound were checked for each sample and corrected if necessary.

#### Metabolite quantification and data normalization.

Peaks were quantified using AUC. In certain instances, biochemical data may have been normalized to an additional factor (e.g., cell counts, total protein as determined by Bradford assay, osmolality) to account for differences in metabolite levels due to differences in the amount of material present in each sample.

### Quantitative metabolite analysis

#### Extraction method.

Tissues (small and large intestinal samples) were weighed and dissolved into methanol (1:4 w/v). The samples were vortexed, homogenized, and centrifuged for 10 minutes with 15,294*g* at 4°C. Then the samples were filtered into HPLC vials and injected to LCMS.

#### LCMS analysis.

The extracted solutions (5 μL) were injected into a UPLC connected to a photodiode array detector (Dionex Ultimate 3000), with a reverse-phase column (ZORBAX Eclipse Plus C18, 100*3.0 mm, 1.8 μm). The mobile phases consisted of phase A DDW with 0.1% formic acid and phase B acetonitrile containing 0.1% formic acid. The gradient was started with 98% A and increased to 30% B in 4 minutes, then increased to 40% B in 1 minute and kept isocratic at 40% B for another 3 minutes. The gradient increased to 50% in 6 minutes, increased to 55% in 4 minutes, and finally increased to 95% in 5 minutes and kept isocratic for 7 minutes. Phase A was returned to 98% A in 3 minutes, and the column was allowed to equilibrate at 98% A for 3 minutes before the next injection. The flow rate was 0.4 mL/min. MS analysis was performed with HESI-II source connected to a Q Exactive Plus Hybrid Quadrupole-Orbitrap Mass Spectrometer from Thermo Fisher Scientific. ESI capillary voltage was set to 3500 V, capillary temperature to 300^°^C, gas temperature to 350°C, and gas flow to 10 mL/min. The mass spectra (*m/z* 100–1500) were acquired in negative ion mode (ESI^–^).

#### Data preprocessing.

Peak determination and peak area integration were performed with QuanBrowser (Thermo Xcalibur, version 4.1.31.9). Autointegration was manually inspected and corrected if necessary. Calibration curves were used for the quantification of each compound. Linear curves were obtained for all compounds with *R*^2^ > 0.99: hippuric acid 0.1–5000 ppb, benzoic acid 500–50,000 ppb, taurocholate 100–50,000 ppb, and deoxycholic acid 0.1–100 ppb.

#### Method validation.

Method validation was performed to determine, limit of detection (LOD), limit of quantitation (LOQ) linearity repeatability, and recovery for each compound.

For interday precision, a mixture of all the samples was prepared and injected as QC at the beginning of the sequence, then after each 10 samples, and at the end of the sequence. The RSDs into QC samples were calculated for each analyte to be less than 6.6%.

For recovery analysis, 3 samples were spiked, extracted, and injected to LCMS. The concentration of each analyte was calculated into the spiked and nonspiked samples, and the recovery was evaluated to be on average 82% for benzoic and hippuric acids, 92% for taurocholate, and 98% for deoxycholic acid.

LOD and LOQ were determined by signal-to-noise ratios higher than 3 and 10, respectively. LOD and LOQ for hippuric acid, deoxycholic acid, and sodium taurocholate was 0.1 ppb. LOD and LOQ for benzoic acid was 500 ppb.

### Metabolomic data analysis

#### Data preprocessing.

The raw metabolome data matrix received from Metabolon contained 31 samples (10 fetus, 11 infant, and 10 pediatric samples) and 833 identified metabolites. The data were preprocessed according to Michonneau et al. (23). Metabolites with missing values in more than half of the samples were filtered out, resulting in 674 remaining metabolites. As the missing values in the metabolome data usually come from the underdetection of true values at the lower limits of MS, missing values were imputed by assigning half of the minimum observed value of each metabolite and addition of a small random noise (Gaussian distribution with mean 0 and variance 100 and then rounded to the nearest integer) to the missing data to avoid the ties across samples that will impair variance estimation in subsequent differential analysis (23). To justify this procedure, we categorized all metabolites into 4 categories: “no missing values,” “low missingness” (missing in 0–4 samples), “medium missingness” (5–9), and “high missingness” (>10). Supplemental Figure 1C shows box plots of log-transformed mean values for metabolites in each category. The results show that metabolites with higher numbers of missing values have significantly lower values, suggesting that missingness likely comes from signal underdetection. Next, data were log-transformed (base 2) and normalized across samples by quantile normalization using the “preprocessCore” package. Supplemental Figure 1D (left plot) shows the box plots of log-transformed values in each sample before normalization with variable intensity levels across samples (with some differences as large as 4-fold), suggesting that proper normalization was needed and successfully achieved (Supplemental Figure 1D; right plot for postnormalization). All downstream analyses were based on the preprocessed normalized data of 31 samples and 674 metabolites after filtering, imputation, log transformation, and quantile normalization.

#### Determination of metabolite clustering.

Gap statistic (24) and prediction strength (25) were used to determine the number of subgroups (Figure 3, A and B) using R package “cluster” and “fpc,” respectively. K-means clustering was performed to generate 3 subgroups (Figure 3C). HCL with Ward linkage and t-SNE (package “Rtsne”) plots were made to determine whether samples could be separated into subgroups by age (Figure 3, D and E).

#### Statistical and bioinformatic analyses.

(a) For differential analysis, we applied the “Limma” package to detect differentially expressed metabolites and pathways. Here we performed 3 pairwise comparisons: fetus versus infant, fetus versus pediatric, and infant versus pediatric. The Limma package was used to calculate *P* values and log-fold changes for each metabolite, and then the Benjamini-Hochberg procedure was applied to correct for multiple testing, to control false discovery rate, and to report *q* value. Volcano plots (Figure 4D and Supplemental Figure 3B) were generated using the “ggplot2” package. (b) For network analysis, following Michonneau et al. (23), we identified similarly behaving chemicals among DE metabolites. The change in the ratio of 2 metabolites between the 2 groups was the parameter of interest. If the change in the ratio of the 2 metabolites was not statistically significant, then there was an edge between them in the network. The network plots (Figure 5 and Supplemental Figure 4) were generated using R package “SARP.compo” (61) and polished in Cytoscape (62). (**c**) Pathway enrichment analysis was conducted using IPA software for metabolome data (QIAGEN; https://www.qiagenbioinformatics.com/products/ingenuitypathway-analysis) (28). This package generates *q* values and enrichment effect sizes (log odds ratio). (d) For machine learning prediction analysis, we applied elastic net method for logistic regression modeling (63) using the “glmnet” package to construct a prediction model with variable (metabolite) selection. The tuning parameter in elastic net (corresponding to the number of selected metabolites) was determined by nested 5-fold cross-validation to avoid overfitting (see Figure 4E and Supplemental Figure 3, C and D, for the 21 selected metabolites and the corresponding coefficients). Receiver operating characteristic curve and AUC were derived based on the unbiased nested cross-validation. To further explore the prediction power of each metabolite, we used each metabolite to build the model (674 separate models in total). Possibly due to the small sample size, the regular generalized linear model had inflated estimation, and the maximum likelihood estimate did not exist. Firth logistic regression (64), a standard way to deal with logistic regression with small sample size (64, 65), was then applied. Leave-one-out CV using Firth logistic regression was used to construct the effect size and AUC values.

### Study approval

All samples were obtained under IRB approvals for fetal samples (18010491, University of Pittsburgh), for infant samples (17070226, University of Pittsburgh), and for pediatric samples (18100039, Edmond and Lily Safra Children’s Hospital). Written informed consent was obtained for fetal and pediatric samples. The infant samples were collected as discarded samples without any identifiable information and as such were deemed as non–human research.

## Author contributions

BM, CM, JT, LW, and DS participated in sample acquisition. YL analyzed the data supervised by GT. OK, SBS, and LO performed the microbial analysis. SK and RS performed the quantitative metabolite analysis. SA, DS, SS, OK, and LK conceived of all the studies. YL and JT wrote the manuscript supervised by LK. All authors have read, edited, and approved the manuscript.

## Supplementary Material

supplemental data

supplemental Table 1

supplemental Table 2

supplemental Table 3

supplemental Table 4

supplemental Table 5

supplemental Table 6

supplemental Table 7

supplemental Table 8

supplemental Table 9

## Figures and Tables

**Table 1:**
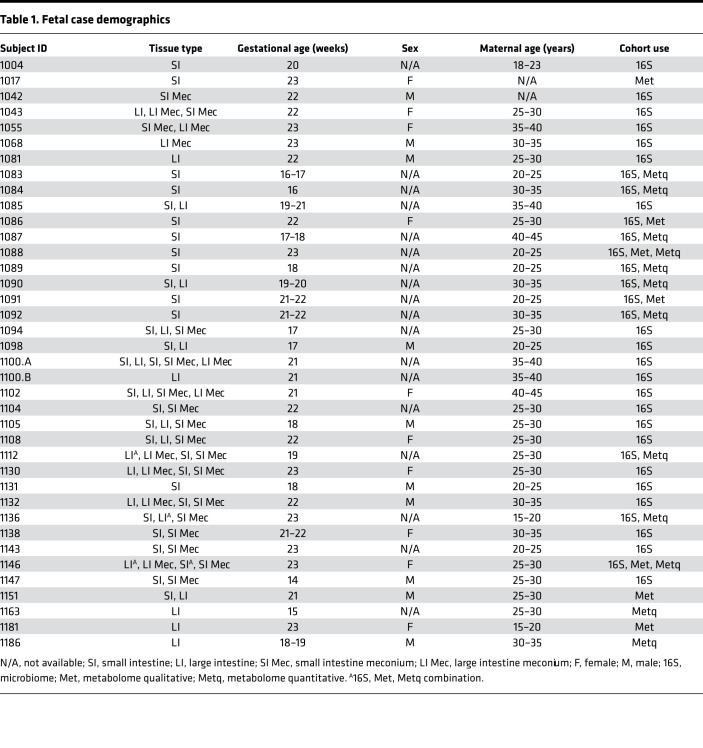
Fetal case demographics

**Table 2:**
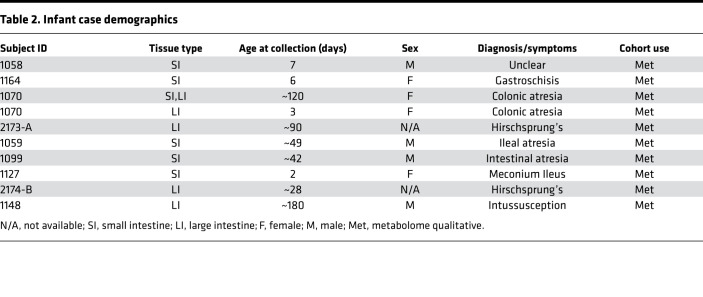
Infant case demographics

**Table 3:**
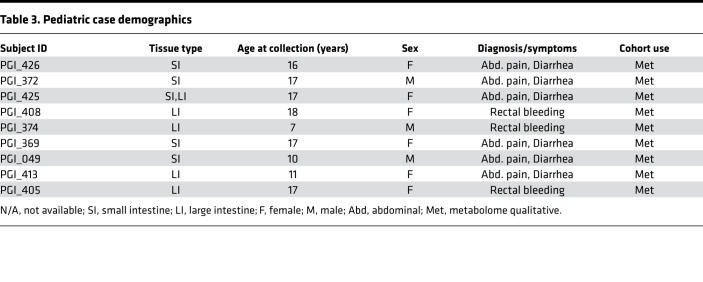
Pediatric case demographics

## Version 1 10/01/2020

In-Press Preview

## Version 2 11/05/2020

Electronic publication
