# Supplementary material for: In utero human intestine harbors unique metabolome, including bacterial metabolites
Source: JCI Insight. 2020 Nov 5;5(21):e138751. doi: 10.1172/jci.insight.138751 (PMC7710283; doi:10.1172/jci.insight.138751)
Supplement: supplemental data [file jciinsight-5-138751-s170.pdf]

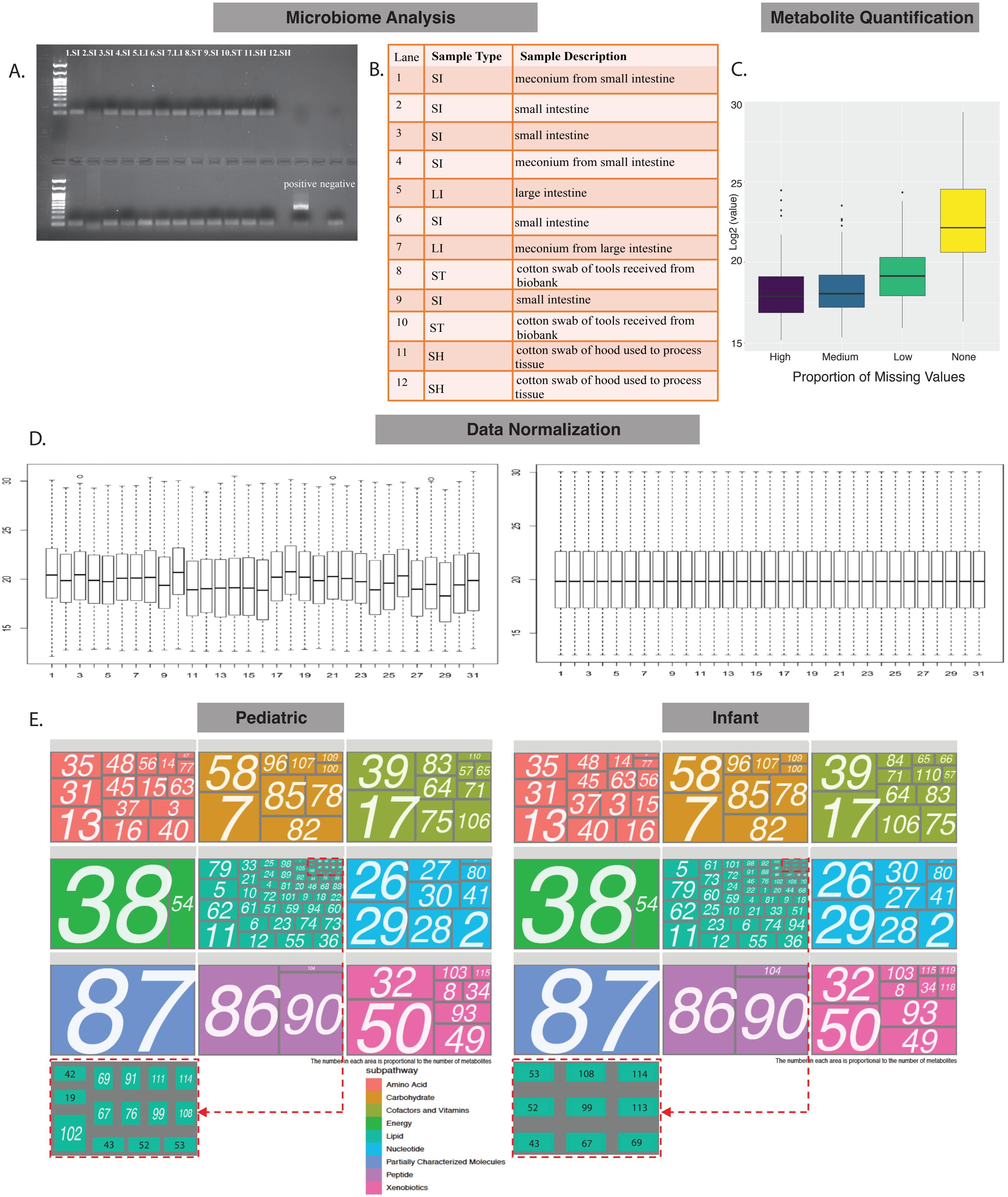

Lane	Sample Type	Sample Description
1	SI	meconium from small intestine
2	SI	small intestine
3	SI	small intestine
4	SI	meconium from small intestine
5	LI	large intestine
6	SI	small intestine
7	LI	meconium from large intestine
8	ST	cotton swab of tools received from biobank
9	SI	small intestine
10	ST	cotton swab of tools received from biobank
11	SH	cotton swab of hood used to process tissue
12	SH	cotton swab of hood used to process tissue

Figure S1. Microbiome and Metabolome of Intestinal Tissue. A representative (A) PCR gel and (B) associated samplesthat were attempted to be amplified for 16S rRNA. (C). Mean values of metabolites by the amount of missing values. (D) Metabolite mean values prior to (left hand side) and after (right hand side) normalization. (E) Treemaps of all expressed metabolites in each group. Associated subpathways in Table S1.

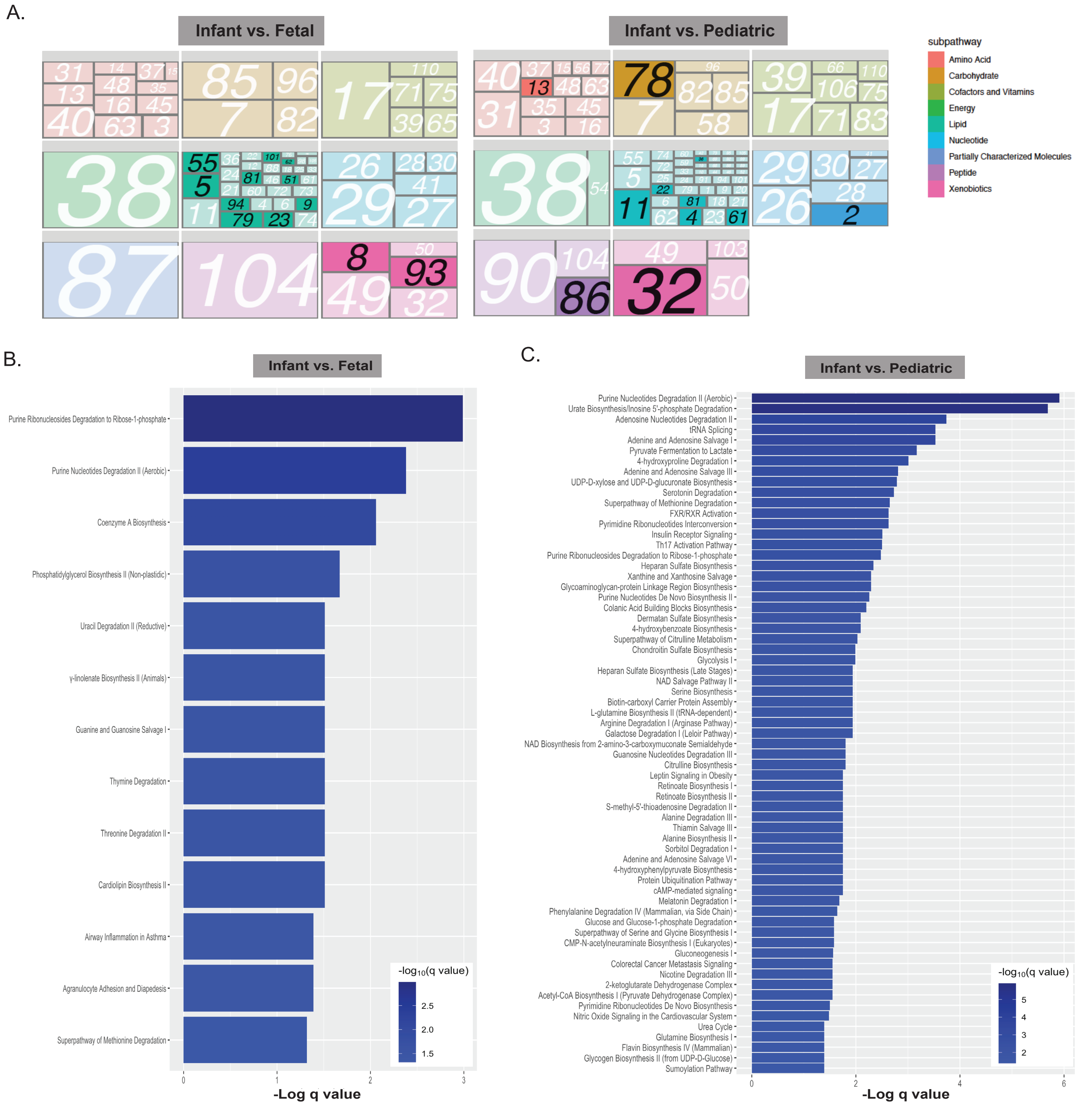

Figure S2. Differential Pathway Expression. (A) Treemap plots by pathway of differentially expressed metabolites. Pathways listed in Table S1. Darker shades and black numbers equally statistically significant differentially expressed pathways by Fisher's exact test (p-value <0.1). (B-C) Integrated pathway analysis of differentially expressed metabolites between noted groups. Bar length is indicative of more significant q-value.

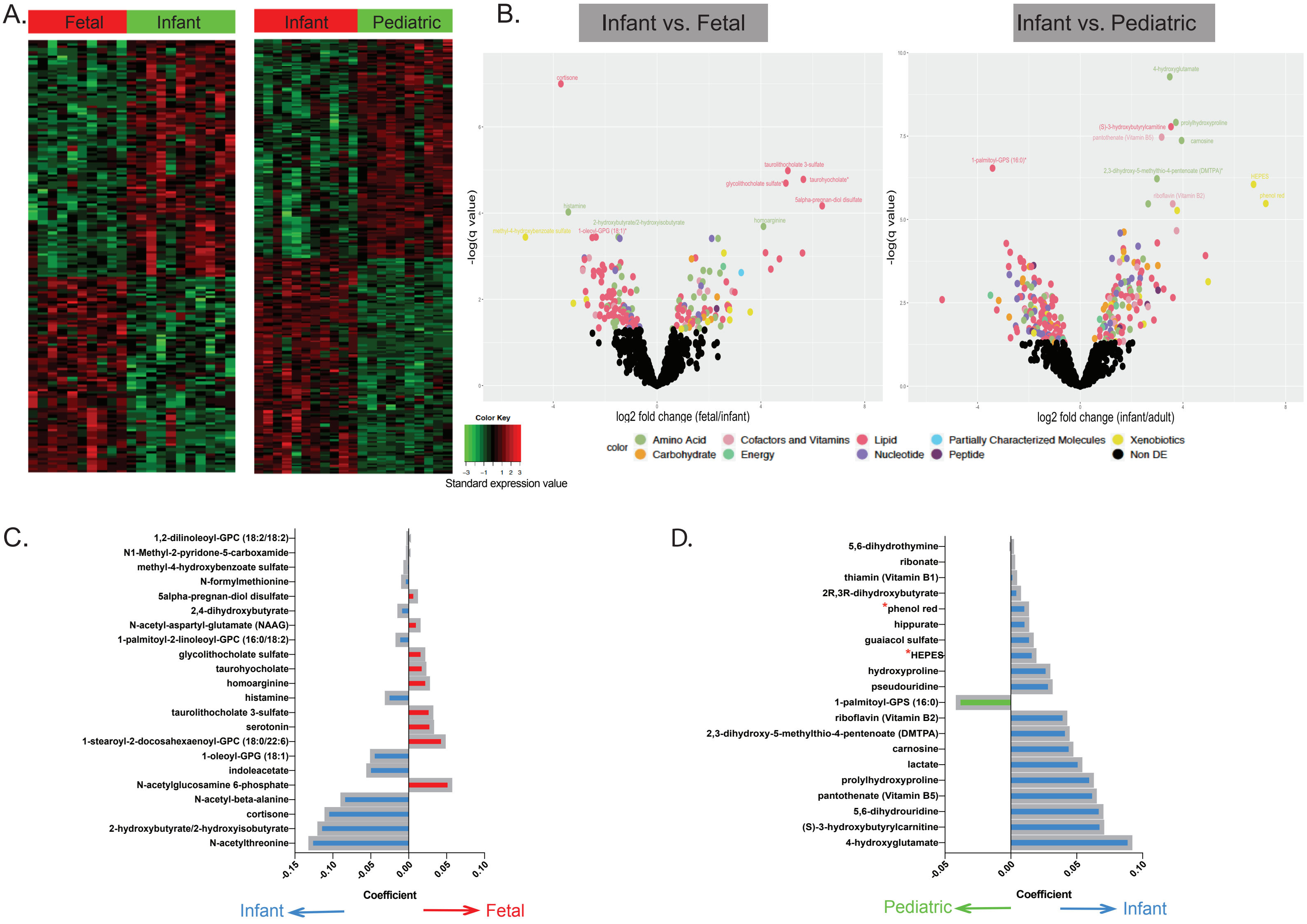

Figure S3. Differential Metabolite Expression. (A) Heatmap of differentailly expressed individual metabolites. List in Table S5. (B) Volcano plots of differentially expressed metabolites. The most differentially expressed metabolites are listed by name. All statistically significantly differentially expressed metabolites (q-value <0.05) are color coded by superpathway. (C-D) Top contributers to elastic net models.

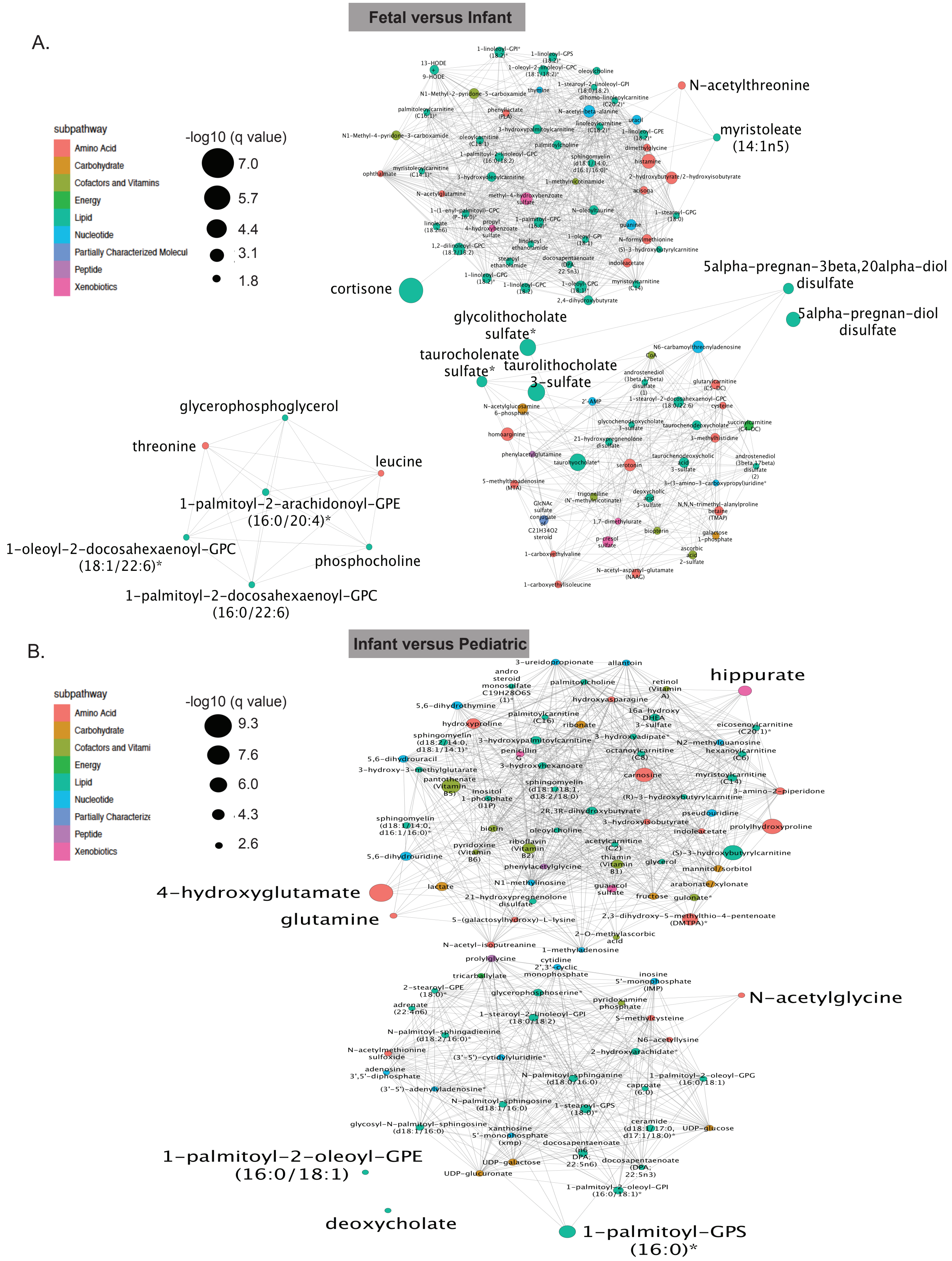

Figure S4. Network analysis. (A-B) Tanimoto network analysis for individual metabolites. Size of circle reflects q-value, color of circle is reflective of superpathway.

A

Type	Description	Purpose
MTRX	Large pool of human plasma maintained by Metabolon that has been characterized extensively.	Assure that all aspects of that Metabolon process are operating within specifications.
CMTRX	Pool created by taking a small aliquot from every customer sample.	Asses the effect of a non-plasma matrix on the Metabolon process and distinguish biologic variablitiy from process variability.
PRCS	Aliquot of UltraPure water.	Process blank used to assess the contribution to compound signals from the process.
SOLV	Aliquot of solvents used in extraction.	Solvent bank used to segregate contamination sources in the solvent.

B

Type	Description	Purpose
RS	Recovery Standard	Assess variability and verify performance of extraction and instrumentation
IS	Internal Standard	Assess variability and performance of instrument.

C

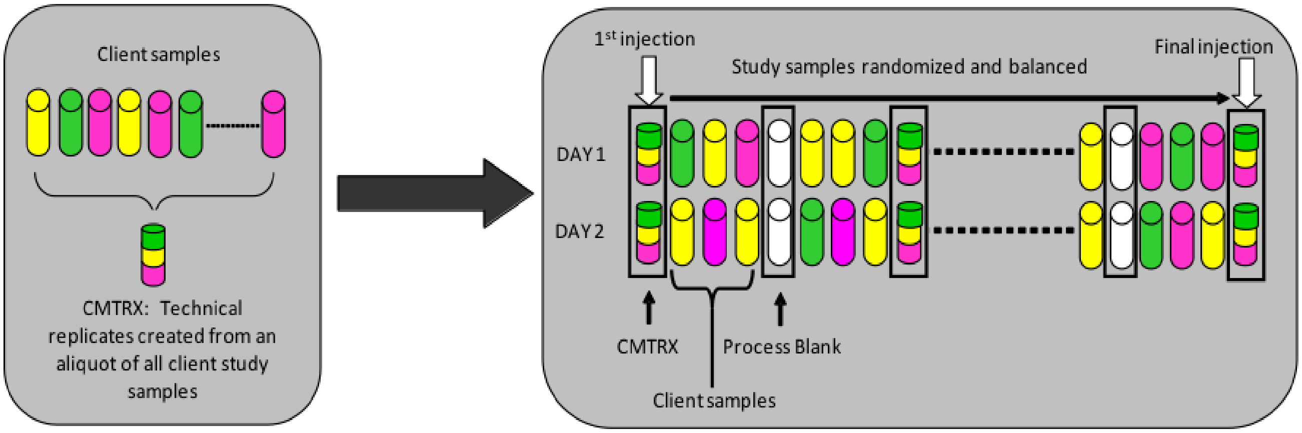

Figure S5. Metabolon quality control (QC) pipeline. A. Description of Metabolon QC samples. B. Description of metabolon AC standards. C. Metabolon biologic and technical control pipeline. A small aliquot of each client sample (colored cylinders) is pooled to create a CMTRX technical replicate sample (multi-colored cylinder), which is then injected periodically throughout the platform run. Variability among consistently detected biochemicals can be used to calculate an estimate of overall
